# Supplementary material for: All-at-once RNA folding with 3D motif prediction framed by evolutionary information
Source: Nat Methods. 2025 Oct 3;22(10):2094–106. doi: 10.1038/s41592-025-02833-w (PMC12510878; doi:10.1038/s41592-025-02833-w)
Supplement: Supplementary file 3 — Source code to run CaCoFold-R3D. Data and results. [file 41592_2025_2833_MOESM3_ESM.gz › supplemental_material/Tables/Table_1.pdf]

RNA family	# seqs	avg length	# bpairs covary/total	Constrained by Covariation Sensitivity (%)	Constrained by Covariation False positives/seq GNRA	Constrained by Covariation False positives/seq K-turn	No covariation used Sensitivity (%)	No covariation used False positives/seq GNRA	No covariation used False positives/seq K-turn
**GNRA motif**									
5S rRNA (RF00001)	50	117	32/34	100	0.14	0	80.65	0.29	0.24
**K-turn motif**									
U3 snoRNA (RF00012)	50	209	13/63	90.3	0.12	0.06	84.95	0.14	0.15
U4 snRNA (RF00015)	50	144	24/28	96.0	0.30	0.02	88.00	0.30	0.10
**Controls**									
6S RNA (RF00013)	50	180	35/52	–	0.02	0.06	–	0.04	0.12
RMF RNA (RF01755)	50	130	1/28	–	0	0	–	0	0
Random Shuffle	20×5	(from all 5 families)		–	0.19	0.12	–	0.07	0.44
Weighted Total				**95.4**	**0.15**	**0.06**	**84.5**	**0.14**	**0.24**
**New K-turns**									
drum bacterial RNA (RF02958)	100	113	22/29	93.0	–	0	89.5	–	0.09
Actinomyces-1 RNA (RF02928)	100	110	11/34	100	–	0.01	98.9	–	0.06
RAGATH-18 RNA (RF03064)	100	71	15/18	97.9	–	0	90.6	–	0.11
HOLDH RNA (RF02997)	10	401	6/95	100	–	0.64	80.0	–	0.60
Weighted Total				**97.8**	–	**0.03**	**92.6**	–	**0.10**

Table 1: **R3D-prototype accuracy on GNRA and K-turn motifs.** The tested RNAs include one of the two tested motifs: the GNRA tetraloop for 5S RNA sequences, and the K-turn internal loop for the U3 and U4 snRNA sequences, or none for the control 6S and RMF RNAs. Sensitivity measures the fraction of motifs identified correctly by the R3D-prototype, where correct identification requires exact matching of the ends of the motif. False positives measure the instances of the tested motif found at the wrong position or instances of the not tested motif. For each RNA, sequences are selected at random from the corresponding alignments. Weighted averages are calculated weighting by the fraction of sequences of a particular RNA type that were analyzed. Several RNAs in the "New K-turns" table also have GNRA loops, thus they cannot be used to estimate GNRA false positives. The location in the alignments of the motifs and covarying base pairs are given in the Supplemental Materials.
